# Hodgkin's Lymphomas: A Tumor Recognized by Its Microenvironment

**DOI:** 10.1155/2011/142395

**Published:** 2010-10-24

**Authors:** S. Montes-Moreno

**Affiliations:** Lymphoma Group, Spanish National Cancer Centre (CNIO), E-28029, Madrid, Spain

## Abstract

Thomas Hodgkin's and Samuel Wilks first recognized Hodgkin disease in the first half of the 19th century. Initially described as lymphogranulomatosis, it was later recognized to be a lymphoid neoplasm derived from B cells and was classified on the basis of its histopathological features. Hodgkin lymphomas are now regarded as encompassing two clearly defined entities according to the WHO classification: nodular lymphocyte-predominant Hodgkin Lymphoma (NLPHL) and classical Hodgkin Lymphoma (CHL). This paper focuses on the current knowledge about the biological features that characterize both NLPHL and CHL, highlighting those relevant to correct pathological diagnosis and those that might be associated with patient outcome.

## 1. Introduction

Hodgkin's disease was first recognized in the first half of the 19th century by Hodgkin [1] and Wilks [2, 3]. Initially described as lymphogranulomatosis, it was later recognized as being a lymphoid neoplasm derived from B cells and classified following the Lukes-Butler scheme [4, 5] on the basis of its histopathological features. This pathological classification was identified early on as the main prognostic marker since the nature of the reactive infiltrate or microenvironment reflected the host response and thus, prognosis [4, 6]. Nowadays Hodgkin's lymphomas is regarded as encompassing two clearly defined entities according to the WHO classification: nodular lymphocyte-predominant Hodgkin lymphoma (NLPHL) and classical Hodgkin lymphoma (CHL) [7–10]. These two entities differ in clinical features and behaviour but, more importantly, in the pathological and biological features of their neoplastic and microenvironmental compartments. 

 The differences in the biological features of the tumours are routinely used in the pathological diagnosis of patients with HL [7, 11] but they can also be exploited as biomarkers of prognosis. This paper focuses on our current knowledge of the biological features that characterize both NLPHL and CHL, highlighting those relevant to correct pathological diagnosis and those that might be associated with patient outcome.

## 2. NLPHL: Usefulness of the Microenvironment in Diagnosis

Nodular lymphocyte-Predominant Hodgkin lymphoma accounts for approximately 5% of all Hodgkin's lymphomas. This type is characterized clinically by a relatively indolent course, a very good response to standard therapies in cases with low-stage disease, but an unfavourable prognosis for advanced stages [9, 12]. Rates of progression to large B cell lymphoma (commonly Diffuse Large B Cell lymphoma (DLBCL), rarely T cell/Histiocyte Rich-like B cell lymphoma (T/HRBCL)) vary according to the series, with a range between 3%–12% of cases [12–16]. 

**Figure 1:**
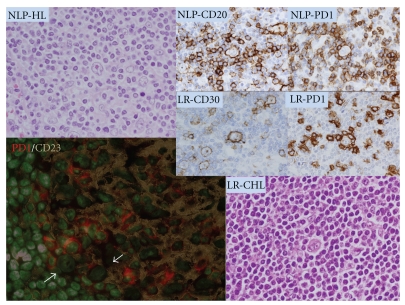
NLP-HL: HE section of a case of Nodular lymphocyte-predominant Hodgkin lymphoma, showing a typical lymphocyte-Predominant (LP) cell positive for CD20 (NLP-CD20) and surrounded by a rim of PD1-positive T cells (NLP-PD1). LR-CHL: HE section of a case of lymphocyte-Rich Classical Hodgkin lymphoma that highlights a typical Reed-Sternberg (R-S) cell with the characteristic phenotype (CD30+, LR-CD30) and surrounded by a rim of PD1-positive T cells (LR-PD1). The pattern is reminiscent of that found in the outer part of the germinal centre where PD1-positive cells surround large B blasts (see arrows in the immunofluorescence capture region of the outer zone of a reactive germinal centre).

 Biologically, NLPHL is a Germinal Centre-(GC-)derived B cell neoplasm [17, 18] that retains an almost complete B Cell program at the transcriptional [19] and phenotypic [8, 20] levels. The characteristic lymphocyte-Predominant (LP) cells exhibit a GC phenotypic profile with expression of GC markers such as BCL6 [11, 21], GCET1 [22] and LMO2 [23], together with expression of transcription factors related to a sustained B cell program such as Oct-2 and BOB.1. Interestingly, the GC-related profile is seen not only in LP cells but also in the surrounding T cells, which characteristically create a rosette-like pattern typical of NLPHL (Figure 1). These rosetting T cells have a Follicular T cell phenotype with expression of CD3/CD4/CD57 [24], bcl6 [21], PD1 [20, 25] and, interestingly, CXCL13, a chemokine that is known to induce B cell homing to lymphoid follicles and that plays a role in the T cell-dependent B cell affinity maturation process [26]. These observations suggest that NLPHL is characterized by a GC phenotype in both LP and T cells. This combination is not found in classical HL (CHL), with the exception of lymphocyte-Rich CHL. In fact, this particular subtype of CHL has a profile intermediate between those of NLPHL and CHL with overexpression of B cell transcription program markers and the presence of a follicular T cell background in a substantial proportion of cases [27] (Figure 1). This is not the case for the other types of CHL in which the B cell program and the germinal centre microenvironment are lost. It is remarkable that this previously described immunohistological pattern (i.e., rosettes of Follicular T cells surrounding large B cells) can also be exploited in the differential diagnosis of NLPHL and T/HRBCL, especially in cases whose morphological features overlap [11, 20, 28].

## 3. Classical Hodgkin Lymphoma (CHL): The Microenvironment as a Prognostic Marker

CHL accounts for 95% of all Hodgkin's lymphomas. This type is characterized by a relative paucity of Reed-Sternberg and Hodgkin neoplastic cells in a background of mixed inflammatory infiltrate by histiocytes, small lymphocytes, eosinophils, neutrophils, plasma cells, fibroblasts and collagen. Depending on the particular combinations of these elements and the specific features of the neoplastic cells, cases may be subclassified into one of four subtypes: nodular sclerosis, mixed cellularity, lymphocyte-rich and lymphocyte-depleted [7]. Initially considered the best predictor of clinical outcome in CHL [4, 6] histological classification *per se* has lost its predictive power, mainly due to the considerable advances in therapeutic regimens [29–31]. These therapeutic improvements have transformed CHL into a curable disease in more than 85% of cases. However, a considerable percentage of patients still fail to respond successfully to current standard therapies. Early identification of these cases has become the main objective of clinical and biological research. In the clinical field it has become apparent that early interim 2-[18F]fluoro-2-deoxy-D-glucose positron emission tomography is a good marker of prognosis and could be used for planning risk-adapted treatment in advanced HL [32]. In the biological field, the extensive study of pathways involved in HL pathogenesis has identified apoptosis deregulation and constitutive NF*κ*B activity as being the factors mainly responsible for tumor unrestricted growth [17, 29, 33]. In particular, constitutive NF*κ*B signalling has been implicated in the activation of upstream receptors [34–38] and in several genetic lesions affecting the NF*κ*B pathway [39–45]. 

**Figure 2:**
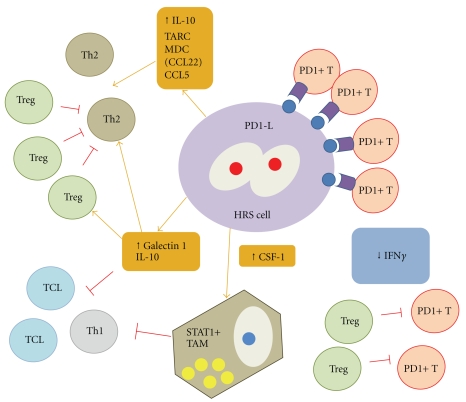
The HRS cell and its cellular microenvironment. Significant interactions with impact in patients outcome are depicted. Increased Galectin1 and IL-10 secreted by HRS (Hodgkin and Reed-Sternberg) cells lead to decreased TCL (T cytotoxic) and Th1 responses, together with a Treg increase which, in turn, suppresses both Th2 and PD1+ T cell activity [46–51]. PD1+CD4+ T cell activity is additionally suppressed by direct interations trough PD1-PD1L immunological synapse (T cell exhaustion) [52]. This leads to a decreased IFN*γ* production. Additionally, increased TAM (Tumor Associated Macrophages) contribute to T cell deletion trough STAT1 signaling pathway [53, 54].

 Together with genetic lesions affecting the neoplastic population, this malignant component is also tightly regulated by interactions with the microenvironment. Of particular interest is the presence of biased T cell populations in HL cases, evidence for a disregulation of T cell immune function. In this sense, a considerable fraction of infiltrating CD4+ T cells are regulatory (Treg) cells which have been shown to display immunosuppressive activity on HL Th2 cells [46]. It has been also shown that together with T regs (recruited due to the secretion of galectin-1 [47, 48]), PD1+ T cells interact with Hodgkin and R-S cells as outlined in the previous section [20, 27, 46, 52, 55]. It has been clearly demonstrated that neoplastic HL cells express PD1-L genes B7-H1 and B7-DC and this expression is induced by EBV latent membrane proteins. Blockade of this immunological synapse leads to restoration of IFN*γ* production by CD4+ T cells in the microenvironment of HL cases and to inhibition of the phophorilation of SHP-2, a mediator of the PD1 signalling pathway. Thus the expression of PD1L genes by HL cells may sustain this immunological synapse that leads to inhibition of IFN*γ* production by TCD4 cells [52]. Blockade of this crosstalk leads to the recovery of T cell antitumoral function thus providing a basis for immunotherapy of HL [56, 57] (Figure 2).

## 4. Translational Biology in CHL

The knowledge based on the biology of CHL has recently become of potential clinical application after the generation of prognostic models of the expression of many of these biological markers. In two very recent papers [58, 59] genome-wide analysis of CHL cases was used to identify gene signatures associated with clinical outcome. These particular gene signatures were validated in routinely processed Formalin-Fixed Paraffin-Embedded (FFPE) samples using RT-PCR [58] or Immunohistochemistry [59], which are both techniques that could be implemented in clinical laboratories. 

 In the study by Sanchez-Espiridion et al. [58] genes were selected on the basis of previous data published by the same group [53] who identified genes involved in the regulation of mitosis and cell growth/apoptosis (TOP2A, RRM2, PCNa, MAD2L1 and CDC2), those expressed by the tumoral cells (as demonstrated by the use of HL cell lines as control), and genes expressed by the microenvironmental compartment (including those expressed by monocyte/macrophages STAT1, ALDH1A1) and T cells (SH2D1A). This particular set of genes was validated by immunohistochemistry in the same series of patients and has recently been confirmed in a new independent retrospective series of HL patients using Real-time PCR [58]. Apoptosis-related genes (BCL2, BCL2L1, CASP3) and cell cycle-associated transcripts (CCNA1, CDC2, CCNA2) are among the markers with confirmed prognostic value, and may provide a rational basis for the use of new therapeutic agents targeting pathways in patients with advanced or refractory CHL. 

 Interestingly, among the pathways associated with worse outcome in CHL in the study by Sánchez-Aguilera et al. [53] monocyte/macrophages prove to be a major determinant of outcome, a finding confirmed in the series of Steidl et al. [59]. These authors identified a gene signature of tumor-associated macrophages that was significantly associated with primary treatment failure. They validated their findings immunohistochemically by quantifying the presence of C68+ macrophages in diagnostic samples, demonstrating that the higher the content of macrophages in the biopsy the greater the risk of progression after primary treatment and relapse following autologous stem-cell transplantation. On the other hand, patients with limited-stage disease and without elevated numbers of CD68+ cells had a long-term disease-specific survival of 100% using current treatment strategies. These data provide further support for the hypothesis that specific tumor microenvironmental components can be used as markers of prognosis in patients with CHL. These results suggests new possibilities for specific therapies that target these cellular components and could be used as adjuvant therapy in patients with poor outcome. Conversely, better identification of good outcome patients at diagnosis could avoid unnecessary overtreatment.

## 5. Conclusions

The molecular dissection of Hodgkin's lymphomas using transcriptional profiling and detailed phenotypic analysis has expanded our view of Hodgkin lymphoma as a disease. First, it has clarified the current classification, establishing NLPHL as a different disease entity from CHL [7] and identifying new markers that can be used in routine practice for this differential diagnosis and with other disease entities. Second, it confirms in a very sophisticated way the pioneer reports [60–62] that assigned to specific microenvironmental cellular components (i.e., macrophages) a role as biomarkers of patient outcome, simultaneously identifying potential new treatment strategies.
